# Thermal Decomposition Kinetics and Compatibility of 3,5-difluoro-2,4,6-trinitroanisole (DFTNAN)

**DOI:** 10.3390/ma14154186

**Published:** 2021-07-27

**Authors:** Fei Hu, Lin-Jian Wang, Wei Zhao, Yu-Cun Liu, Su-Ming Jing, Ping Liu, Jin-Xuan He

**Affiliations:** 1 School of Environment and Safety Engineering, North University of China, Taiyuan 030051, China; B1914079@st.nuc.edu.cn (F.H.); S1914007@st.nuc.edu.cn (L.-J.W.); S1814082@st.nuc.edu.cn (W.Z.); 2 Chongqing Hongyu Precision Industry Group Co., Ltd., Chongqing 402760, China; 18234138606@sohu.com; 3 Science and Technology on Aerospace Chemical Power Laboratory, Hubei Institute of Aerospace Chemotechnology, Xiangyang 441003, China

**Keywords:** DFTNAN, cast explosives, thermal behavior, compatibility

## Abstract

In this paper, the thermal decomposition behavior of 3,5-difluoro-2,4,6-trinitroanisole (DFTNAN) was studied by differential scanning calorimetry (DSC) and thermogravimetry (TG) by using different heating rates (2, 5, 10, 15 °C·min^−1^). Subsequently, the kinetic and thermodynamic parameters of non-isothermal thermal decomposition of DFTNAN were calculated. The critical temperature of thermal explosion ($T_{b}$) and self-accelerating decomposition temperature ($T_{\mathrm{ASDT}}$) were determined to be 249.03 °C and 226.33 °C, respectively. The compatibility of DFTNAN with a number of high explosives (cyclo-1,3,5-trimethylene-2,4,6-trinitramine (RDX), 1,3,5,7-tetranitro-1,3,5,7-tetrazocine (HMX), 2,4,6,8,10,12-hexanitro-2,4,6,8,10,12-hexaaza-tetracyclo-[5.5.0.05,9.03,11]-dodecane (CL-20) and dihydroxylammonium 5,5’-bistetrazole-1,1’-diolate (TKX-50)) was studied at different mass ratios using DSC. The criteria to judge the compatibility between the materials were based on a standardization agreement (STANAG 4147). The thermodynamic study results revealed that DFTNAN possessed superior thermal safety and stability. The experimental of compatibility results indicated that the mass ratios of the high explosives in the DFTNAN/RDX, DFTNAN/HMX and DFTNAN/CL-20 compositions more than 40%, 60% and 70% exhibited good compatibility, whereas DFTNAN/TKX-50 demonstrated poor compatibility.

## 1. Introduction

The melt-cast explosive is a mixed explosive material that can be cast in the molten state [1]. As the melt-cast explosive can adapt to various shapes and has a comprehensive performance, it represents a military grade mixed explosive. It is widely used in the loading of grenades, armor piercing bombs, aerial bombs, mine and missile warheads [2,3], etc. The liquid phase carrier of the melt-cast explosive is a key component. As a special energetic binder [4], the melt-casting carrier can not only improve the safety of high-energy explosives alone [5], but also enhance the structural integrity and mechanical properties of propellant and ammunition charges [6,7,8]. Compared with ordinary polymer binder, it has environmental friendliness and higher economic benefits [9,10]. For long, 2,4,6-trinitrotoluene (TNT) has been widely used as a carrier of the melt-cast explosive. With the requirement of low vulnerability of the military explosives put forward in the 1970s, TNT no longer meets the requirements for developing the modern weapons and ammunition [11]. In addition, TNT as the liquid carrier of the explosives has been improved by adding other high-energy materials to form a low eutectic material. Overall, an effective synthesis and design of the liquid phase carrier is needed to replace TNT [12].

Currently, a number of melt-cast explosive carriers which are expected to replace TNT have been synthesized, such as 2,4-dinitrobenzene (DNAN), 3,4-dinitropyrazole (DNP) [13], 1-methyl-3,4,5-trinitropyrazole (MTNP) [14], 1-methyl-2,4,5-trinitroimidazole (MTNI) [15,16], 3,4-dinitrofurazanfuroxan (DNTF) [17], 1-methyl-3,5-dinitropyrazole (DNMT) [18], 1-nitroamino-2,3-dinitrate propane (NG-N1) [19], etc. However, these suffer from different shortcomings and do not meet the requirements of widespread application.

**Figure 1:**
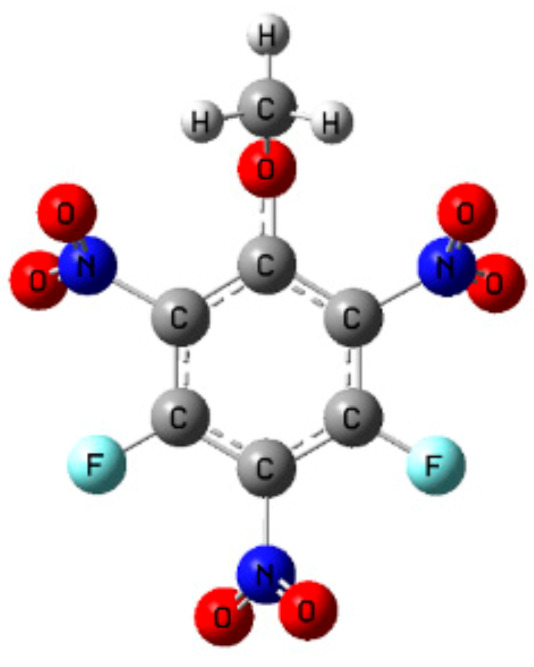
Chemical structure of DFTNAN.

2,4,6-trinitro-3,5-difluorophenyl methyl ether (DFTNAN) is a new class of fusion casting carrier [20]. Its structure is shown in Figure 1. DFTNAN possesses high density (1.81 g·cm^−3^), low melting point (82 °C), high decomposition temperature (285 °C) and low sensitivity to external stimuli [20]. In addition, it demonstrates a superior detonation velocity and detonation pressure. Its thermal behavior and compatibility have not been reported in the literature so far, thus, requiring in-depth analysis.

Therefore, in this study, the thermal decomposition behavior of DFTNAN was studied by differential scanning calorimetry (DSC) and thermogravimetry (TG). The non-isothermal thermal decomposition kinetics of DFTNAN was explored by using the Kissinger and Ozawa methods. The apparent activation energy and pre-exponential factor were obtained subsequently. At the same time, the thermodynamic parameters, including the peak decomposition temperature, self-accelerating decomposition temperature and thermal explosion critical temperature were determined, along with evaluating the thermal safety of DFTNAN. Based on the STANAG 4147 standard, the compatibility of DFTNAN with cyclo-1,3,5-trimethylene-2,4,6-trinitramine (RDX), 1,3,5,7-tetranitro-1,3,5,7-tetrazocine (HMX), 2,4,6,8,10,12-hexanitro-2,4,6,8,10,12-hexaaza-tetracyclo-[5.5.0.05,9.03,11]-dodecane (CL-20) and dihydroxylammonium 5,5’-bistetrazole-1,1’-diolate (TKX-50) composite explosives were evaluated at different mass ratios by DSC. The findings obtained in this study are expected to act as a benchmark for the application of DFTNAN in melt-casting explosives.

## 2. Experimental

### 2.1. Materials

DFTNAN was prepared in the lab with a purity of 98%.

The solid high energy explosives, technical grade RDX, HMX, CL-20 and TKX-50 with a purity of more than 97% were supplied by Qingyang Chemical Industry Corporation (Liaoning, China), Gansu Yinguang Chemical Industry Group (Gansu China, North Industries Group (Beijing, China) and Liming Research Institute of Chemical Industry (Henan, China), respectively.

### 2.2. Preparation of Samples

Preparation of the test sample for the thermal decomposition analysis: DFTNAN was dried in an explosion-proof oven at 60 °C for 4 h, followed by grinding with an agate mortar and passing through 120 mesh screen.

Preparation of the sample for the compatibility test: The binary mixtures of DFTNAN with RDX, HMX, CL-20 and TKX-50 were prepared at different mass ratios, as shown in Table 1.

The components were dried in an explosion-proof oven at 60 °C for 4 h. Afterwards, DFTNAN was completely melted, and the solid high explosive (RDX/HMX/CL-20/TKX-50) was added in batches as per the relation shown in Table 1. The capillary stirring was employed during the process. After the addition of the explosive, the mixture was stirred for 5 min, followed by cooling to room temperature. The solidified casting explosives with different compositions were ground into powder form with an agate mortar.

### 2.3. Characterization

Thermal stability: The thermal decomposition behavior the samples were determined in the temperature range 50–350 °C using the heating rates of 2, 5, 10 and 15 °C·min^−1^ by, respectively, employing HCT-1 (HENVEN Instruments, Beijing, China) and TAG 5500 (TA Instruments, New Castle, DE, USA) under N_2_ atmosphere. For DSC, about 1.0~1.5 mg sample mass was taken in the aluminum pans with cover. On the other hand, the TG sample weighing about 5.0 mg was taken in a ceramic crucible.

Compatibility: The samples (1.0 mg) taken in the aluminum pans with cover were characterized by using HCT-1 in the temperature range 50 °C to 350 °C at a heating rate of 5 °C·min^−1^ from under N_2_ atmosphere. For assessing the compatibility, the peak decomposition of DFTNAN was compared with the peak decomposition of the mixtures (DNTNAN and RDX/HMX/CL-20/TKX-50).

## 3. Results and Discussion

### 3.1. Thermal Decomposition Behavior of DFTNAN

#### 3.1.1. DSC Analysis

**Figure 2:**
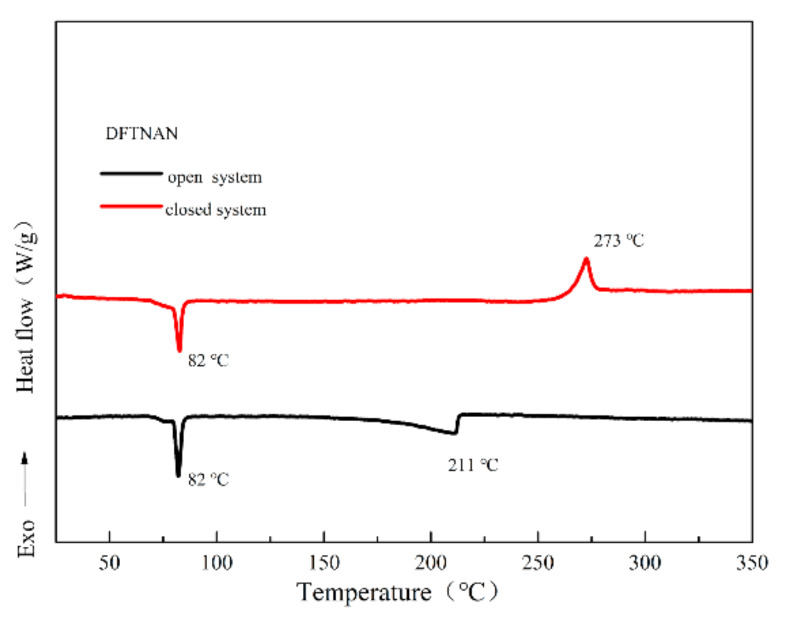
DSC curves for DFTNAN at heating rate 5 °C min^−1^ (under different conditions).

The DSC curves of DFTNAN under different conditions acquired using a heating rate of 5 °C·min^−1^ are presented in Figure 2. In the open condition, the DSC curve of DFTNAN demonstrated two endothermic peaks at 82 °C and 211 °C, which indicated the melting and evaporation processes, respectively. However in the closed condition, an endothermic peak and an exothermic peak were observed, which corresponded to the melting at 82 °C and decomposition at 273 °C. In the open condition, no decomposition was detected up to 273 °C, as DFTNAN reached the boiling point before reaching the decomposition temperature.

**Figure 3:**
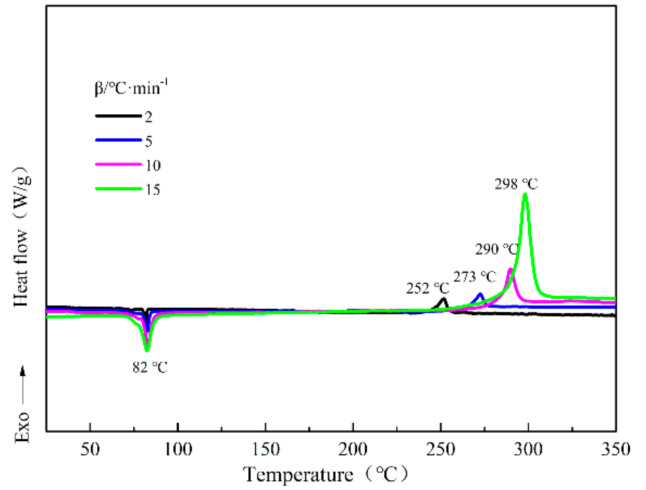
DSC curves for DFTANA at different heating rates of 2, 5, 10 and 15 °C min^−1^.

The DSC curves of DFTNAN acquired using the heating rates of 2, 5, 10 and 15 °C·min^−1^ are shown in Figure 3. As shown, the temperature of the decomposition peak (*T*_p_) increased on enhancing the heating rate, with the peak becoming wider. However, the temperature of the melting peak exhibited only a slight change. The observed shifting of the exothermic peak to the high temperature might be attributed to an increment in the thermal effect per unit time and temperature difference. Further, the reduction in the reaction time and incomplete decomposition reaction on enhancing the heating rate also contributed to this effect [21]. Thus, the synthesis leads to a lag in the heat release. The epitaxial starting temperature (*T*_eo_), peak temperature (*T*_p_) and end decomposition temperature (*T*_f_) of DFTNAN at different heating rates are listed in Table 2.

#### 3.1.2. TG Analysis

**Figure 4:**
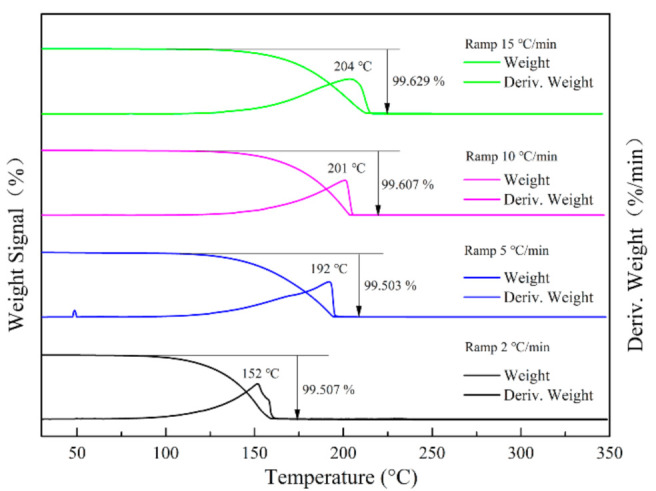
TG curves for DFTANA at different heating rates of 2, 5, 10 and 15 °C min^−1^.

The TG-DTG curves of DFTNAN at various heating rates are shown in Figure 4. Only one step decomposition was observed in the TG curves, and the mass loss rate was close to 100%. It indicated that the decomposition of DFTNAN was a complete and continuous process. On increasing the heating rate, the temperature T_V_ at the maximum mass loss rate increased, as expected. The observed behavior was consistent with the trend observed in the DSC curves: on increasing the heating rate, *T*_p_ and *T*_V_ at the maximum mass loss rate exhibit an increase. A significant difference was observed between the *T*_V_ (DTG) and *T*_p_ (DSC) values at different heating rates, which was due to the fact that the DSC analysis was performed in the closed conditions, and the TG analysis was carried out in the open condition.

#### 3.1.3. Non-Isothermal Decomposition Kinetics

In order to obtain the apparent activation energy$\;(E_{k}$) and pre-exponential factor$\;\left(A_{k}\right)\;$for the thermal decomposition of DFTNAN, the Ozawa and Kissinger methods were applied. The equations for the Kissinger (1) and Ozawa (2) methods can be written as follows [22,23]:

(1) $$\ln\frac{\beta_{i}}{T_{\mathrm{pi}}^{2}}=\ln\frac{A_{k}R}{E_{k}}-\frac{E_{k}}{{RT}_{\mathrm{pi}}},\;i=1,2,\ldots ,4$$

(2) $${\log\beta }_{i}=\log\left(\frac{A_{k}E_{o}}{Rg\alpha }\right)-2.315-\frac{0{.4567E}_{o}}{{RT}_{\mathrm{pi}}},i=1,2,3,\ldots$$

where, *R* is the gas constant (8.314 J·mol^−1^·K^−1^), $\beta_{i}$ is the linear heating rate (K·min^−1^) and $T_{\mathrm{pi}}$ corresponds to the peak decomposition temperature in case the heating rate is $\beta_{i}$ (K).

As per the Kissinger and Ozawa equations, the values of $\ln\frac{\beta_{i}}{T_{\mathrm{pi}}^{2}}$ and $\log\beta_{i}$ were plotted against $\frac{1}{T_{\mathrm{pi}}}$, respectively. The regression equation and correlation coefficient were subsequently obtained, where the $E_{k}$ and $E_{o}$ values were determined from the slope, and the values of $A_{k}$ were obtained from the intercept, as shown in Table 3. As observed, the apparent activation energy of DFTNAN obtained by the Kissinger method was in good agreement with the energy obtained by the Ozawa method. Further, the linear correlation coefficients were noted to be close to 1, indicating that the calculation results were reliable.

#### 3.1.4. Thermodynamic Parameters of Activation Reaction

The entropy ($\Delta S^{\neq }$), enthalpy ($\Delta H^{\neq }$) and Gibbs free energy ($\Delta G^{\neq }$) of activation of the exothermic decomposition of DFTNAN were calculated according to Equations (3)–(5) [24].

(3) $$A=\frac{kT_{p}}{h}\exp\left(\frac{\Delta S^{\neq }}{R}\right)$$

(4) $$\Delta H^{\neq }{=E}_{k}{-RT}_{p}$$

(5) $$\Delta G^{\neq }=\Delta H^{\neq }{-T}_{p}\Delta S^{\neq }$$

where, *k* is the Boltzmann’s constant (1.3807 × 10^−23^ J·K^−1^), and *h* is the Planck’s constant (6.625 × 10^−34^ J·s^−1^).

By using the calculated data, $\Delta S^{\neq }$, $\Delta H^{\neq }$ and $\Delta G^{\neq }$ at *T*_p_ were calculated. The $\Delta S^{\neq }$ value was noted to be negative, as per the transition state theory, which indicated that the order of the transition state structure was increased. On the other hand, the $\Delta H^{\neq }$ and $\Delta G^{\neq }$ values were observed to be positive, which indicated a non-spontaneous process related to the introduction of heat, thereby revealing the superior thermal stability of DFTNAN.

#### 3.1.5. Thermal Safety Parameters

The critical temperature of thermal explosion ($T_{b}$) is a vital parameter to evaluate the safety of the materials and ascertain the transformation from thermal decomposition to thermal explosion [25]. On the other hand, the self-accelerating decomposition temperature ($T_{\mathrm{ASDT}}$) represents the maximum allowable ambient temperature for practical application.

For DFTNAN, the polynomial regression method ($T_{\mathrm{ei}}=T_{\mathrm{eo}}+\alpha \beta_{i}+b\beta_{i}^{2}+c\beta_{i}^{3}$) was used. For β = 0, the epitaxial starting temperature $T_{\mathrm{eo}}$ was obtained more accurately and $T_{\mathrm{ASDT}}=T_{\mathrm{eo}}$ [26]. The $T_{b}$ value was obtained by substituting $T_{\mathrm{eo}}$ in Equation (6) [27].

(6) $$T_{b}=\left[E-{\left(E^{2}-4ERT_{\mathrm{eo}}\right)}^{\frac{1}{2}}\right]/2R$$

The values of $T_{\mathrm{eo}}$ ($T_{\mathrm{ASDT}}$) and $T_{b}$ were determined to be 226.33 °C and 249.03 °C, respectively. Therefore, the high epitaxial starting temperature and critical temperature of thermal explosion of DFTNAN revealed its superior thermal safety and stability at room temperature.

### 3.2. Compatibility

The compatibility is closely related to the safety and reliability of the explosives [28]. The compatibility of DFTNAN with the high energy solid explosives was evaluated by determining the maximum peak temperature difference Δ*T*_p_ from DSC, according to the STANAG 4147 criterion [29] (Table 4).

**Figure 5:**
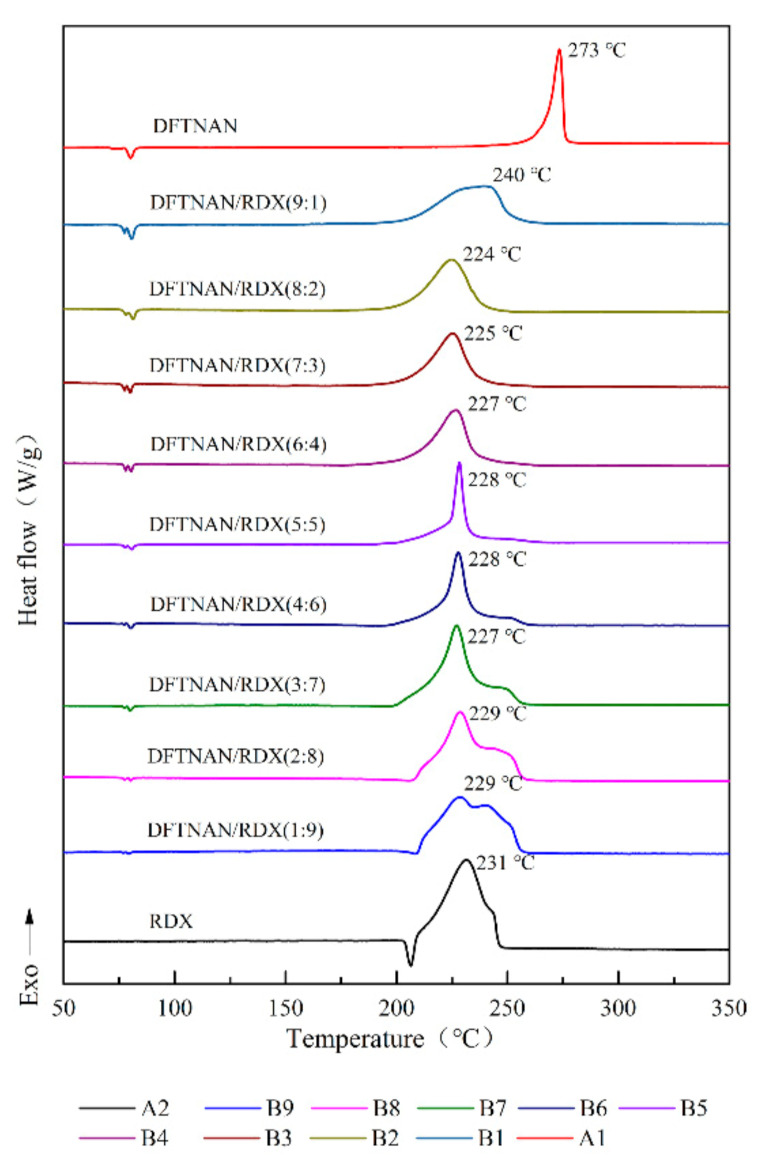
DSC curves of DFTNAN, RDX and mixtures with different ratios.

**Figure 6:**
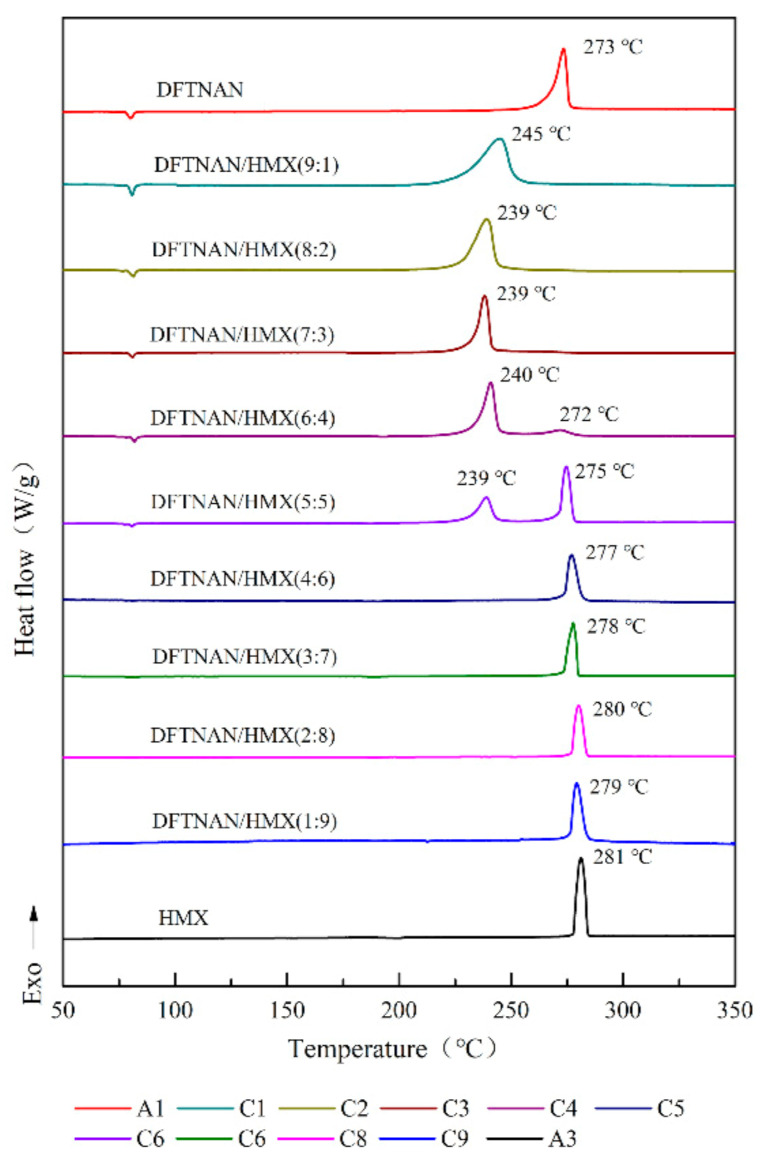
DSC curves of DFTNAN, HMX and mixtures with different ratios.

**Figure 7:**
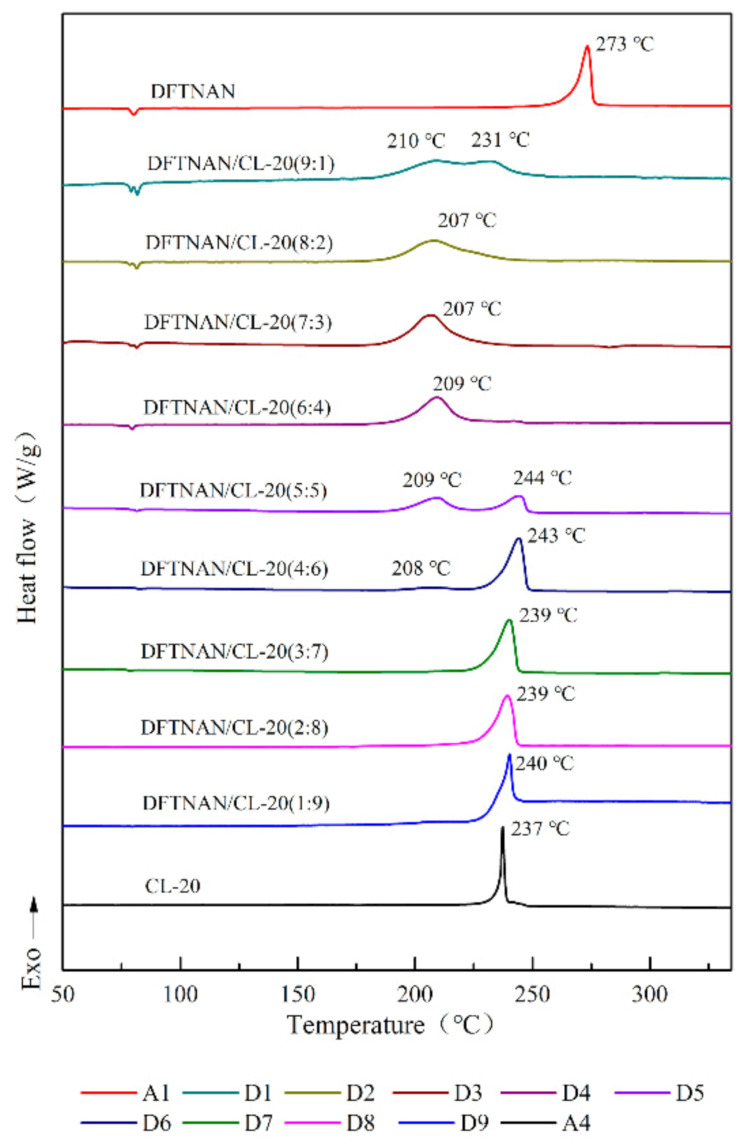
DSC curves of DFTNAN, CL-20 and mixtures with different ratios.

**Figure 8:**
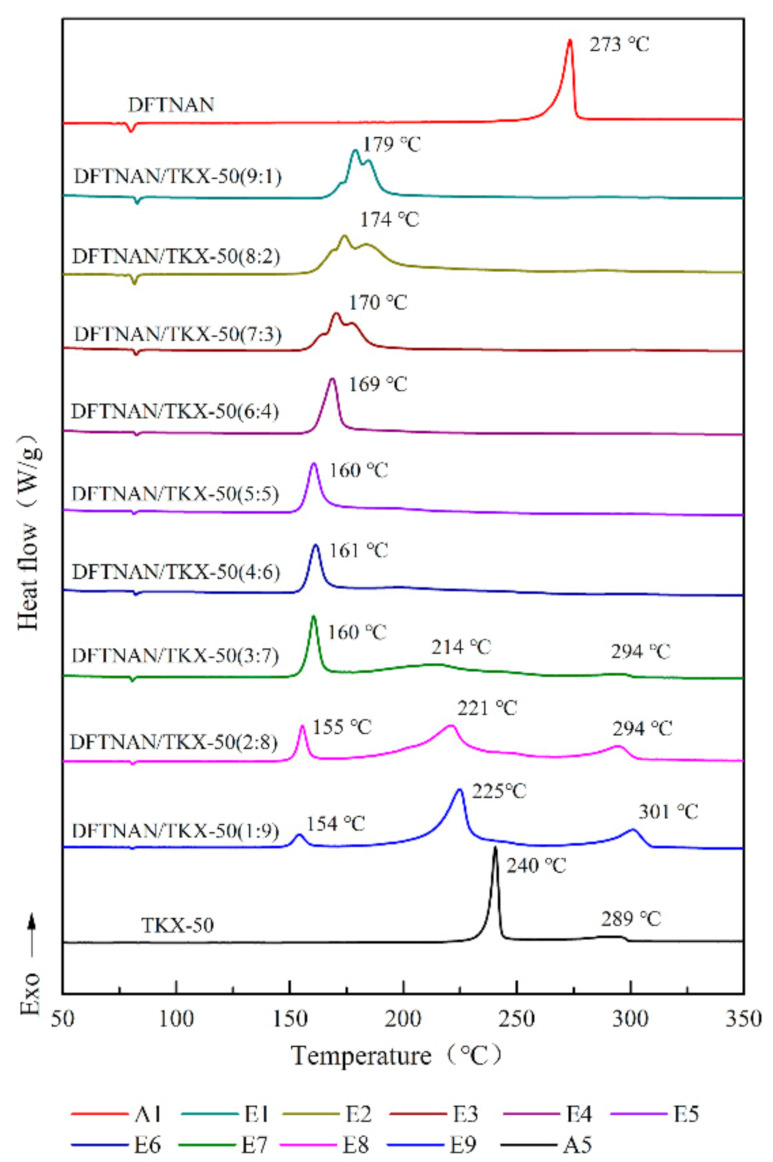
DSC curves of DFTNAN, TKX-50 and mixtures with different ratios.

The DSC curves of pure DFTNAN (A1), pure RDX (A2), pure HMX (A3), pure CL-20 (A4), pure TKX-50 (A5), DFTNAN/RDX (B1–B9), DFTNAN/HMX (C1~C9), DFTNAN/CL-20 (D1~D9) and DFTNAN/TKX-50 (E1–E9) in the closed condition at a heating rate of 5 °C·min^−1^ are presented in Figure 5, Figure 6, Figure 7 and Figure 8. The corresponding evaluated compatibility values are summarized in Table 5.

As observed, the addition of the high explosives to DFTNAN significantly reduced the decomposition peak temperature of the mixed samples, especially on adding TKX-50. The reason for the observed behavior might be attributed to the partial solubility of the N-nitramines explosives (RDX, HMX and CL-20) in DFTNAN. On increasing the temperature, a part of the N-nitramines explosive was dissolved in the melting DFTNAN. Due to the advanced decomposition of DFTNAN induced by the liquid N-nitramines explosive, the peak decomposition temperature of the DTNAN/N-nitramines explosive composition decreased correspondingly. However, TKX-50 is an energetic ionic salt, and the proton transfer may reduce the reaction barrier under thermal stimulation [30], leading to the formation of a molten mixture with melting DFTNAN. The led to the decomposition temperature of DFTNAN/TKX-50 decrease significantly.

The solubility of the N-nitramines explosives in DFTNAN is limited. On enhancing the N-nitramines explosive content, the induction effect of the liquid N-nitramines explosive decreased. The peak decomposition temperature of the mixed samples was correspondingly affected. The DSC curves of the DFTNAN/N-nitramines composition are shown in Figure 5, Figure 6 and Figure 7.

#### 3.2.1. DFTNAN/RDX

As observed in Figure 5, there were an exothermic peak in DSC curves of samples B1–B9. The peak temperature observed in B1 sample was higher than A2 (237 °C), which corresponded to the decomposition of DFTNAN. The Δ*T*_p_ values for B1 sample was 9 °C. The peak temperatures of the B2–B9 samples were lower than A2 (237 °C), which was ascribed to the decomposition of RDX. Apart from B2 and B3 samples which had the Δ*T*_p_ values of 7 and 6 °C, all other samples did not exceed 4 °C. The Δ*T*_p_ values of the samples indicated that RDX was compatible with DFTNAN, except for the mass ratio of 10:90, 20:80 and 30:70 exhibiting moderate incompatibility.

As reported in literature [31], the melting point of RDX is 207 °C. The DSC curve for the composition with a high content of DFTNAN exhibited no melting peak of RDX, whereas the melting peak appeared on increasing the RDX content. This was due to the reason that the low RDX content led to the partial dissolution of RDX through an autocatalytic reaction, thus, speeding up the decomposition rate of the whole system. The melting process was carried out together with the decomposition process, thus, that the melting peak of RDX did not appear in the DSC curve.

#### 3.2.2. DFTNAN/HMX

There was a single exothermic peak in DSC curves of samples C1–C3, which was ascribed to the decomposition of DFTNAN. The peak temperatures of these samples were noted to be lower than A1. The Δ*T*_p_ values for the C1–C3 samples were more than 20 °C, while the result indicated these compositions to be incompatible. On further increasing the HMX content, the peak decomposition temperature of the mixed samples was correspondingly affected.

In Figure 6, the DSC curves for the samples C4 and C5 exhibited two exothermic peaks, which corresponded to the decomposition of DFTNAN and HMX. The first peak became weaker, whereas the second peak became stronger on increasing the HMX content. The *T*_p_ values for the first and second peaks for the samples were determined to be 33 and 34 °C or 9 and 12 °C, respectively. As the HMX content was more than 60%, the DSC curves for the C6–C9 samples displayed only a single exothermic peak, which was ascribed to the decomposition of HMX. The Δ*T*_p_ values for the samples were noted to be <4 °C. This indicated that the DFTNAN/HMX composition with the mass ratios of 10:90, 20:80, 30:70 and 90:10 exhibited optimal compatibility. It should be noted that the melting endothermic peak of A3 was not observed due to the low heating rate [32].

#### 3.2.3. DFTNAN/CL-20

In Figure 7, except for the D1 sample which had two partially overlapping exothermic peak, the DSC curves of D2–D4 samples displayed one exothermic peak, which was attributed to the decomposition of DFTNAN. The peak temperatures of these samples were noted to be lower than A1. The Δ*T*_p_ values for the D1–D4 samples were more than 20 °C, while the result indicated these compositions to be incompatible.

On increasing the CL-20 content, two exothermic peaks were observed in the DSC curves of D5 and D6 sample, which correspond to the decompositions of CL-20 and DFTNAN, respectively. The first peak became weaker, whereas the second peak became stronger on increasing the CL-20 content. It indicated that the DFTNAN/CL-20 mixture with the mass ratios of 50:50 and 40:60 were incompatible.

For the DFTNAN/CL-20 mixture with mass ratio of 30:70, 20:80 and 10:90, only a single exothermic peak was noted, which corresponded to the decomposition of CL-20. The Δ*T*_p_ values of the samples indicated that CL-20 was compatible with DFTNAN.

#### 3.2.4. DFTNAN/TKX-50

In Figure 8, for the mass ratios of the high explosives in the DFTNAN/TKX-50 compositions less than 60%, only a single exothermic peak was noted, which was ascribed to the decomposition of DFTNAN. The peak temperatures of these samples were noted to be lower than A1. The Δ*T*_p_ values for the E1–E6 samples were more than 20 °C, while the result indicated these compositions to be incompatible.

On further increasing the TKX-50 content, three exothermic peaks were observed in the DSC curves of E7, E8 and E9. Among these, the first peak was attributed to DFTNAN, and the Δ*T*_p_ values were noted to be 113, 118 and 119 °C, respectively. The second and third peaks were attributed to TKX-50 and were lower than the peak temperature of A5 (26, 19, 15 °C and 5, 5, 12 °C, respectively). This indicated that TKX-50 was incompatible with DFTNAN, and the thermal stability of pure explosive was reduced by mixing DFTNAN with TKX-50. It should be noted that the peak temperature of the third exothermic peak of E8 was higher than that of the second decomposition peak of TKX-50. Thus, it could be suggested that the addition of DFTNAN affected the thermal decomposition characteristics of TKX-50.

## 4. Conclusions

The thermal decomposition behavior of DFTNAN was investigated by DSC and TG using different heating rates (2, 5, 10 and 15 °C·min^−1^). The peak decomposition temperature (*T*_p_) was observed in the range 252–298 °C, whereas the melting temperature was about 82 °C. The TG curves exhibited a single step decomposition for different heating rates, and the mass loss rate was close to 100%.

By applying the DSC data, the kinetic and thermodynamic thermal decomposition parameters, as well as the thermal safety parameters of DFTNAN, were calculated. The apparent activation energy ($E_{k,0}$) and pre-exponential factor ($A_{k}$)for the thermal decomposition of DFTNAN were estimated by the Ozawa and Kissinger methods. The values of $E_{k}$, $A_{k}$ and $E_{0}$ were determined to be 97.93 kJ·mol^−1^, 4.73 × 10^8^ min^−1^ and 101.78 kJ·mol^−1^, respectively.

The entropy ($\Delta S^{\neq }$), enthalpy ($\Delta H^{\neq }$) and Gibbs free energy ($\Delta G^{\neq }$) of activation at *T*_p_ as well as the critical temperature of thermal explosion ($T_{b}$) and self-accelerating decomposition temperature ($T_{ASDT}$) were calculated as follows: $\Delta S^{\neq }$ = −92.10 kJ·mol^−1^·K^−1^, $\Delta H^{\neq }$ = 95.28 kJ·mol^−1^, $\Delta G^{\neq }$ = 146.04 kJ·mol^−1^, $T_{\mathrm{ASDT}}$ = 226.33 °C and $T_{b}$ = 249.03 °C. The observed thermal parameters confirmed that DFTNAN possessed superior thermal safety and stability. The research results further confirm the potential of DFTNAN as a substitute for melt-casting carrier, and provide a basis for the development, production and storage of explosives.

For the mass ratios of the high explosives in the DFTNAN/RDX mixture no less than 40%, the DFTNAN and RDX have good compatibility. The DFTNAN/HMX mixture with the mass ratios of 40:60, 30:70, 20:80 and 10:90 revealed optimal compatibility. The DFTNAN/CL-20 mixture with the mass ratios of 30:70, 20:80 and 10:90 have good compatibility. On the other hand, DFTNAN was noted to be incompatible with TKX-50. As some basic data, further research on the formula design and cocrystal explosive of DFTNAN/HMX, DFTNAN/RDX and DFTNAN/CL-20 will be carried out, focusing on the study of DFTNAN/CL-20 based casting formula.

## Figures and Tables

**Table 1:** Test samples information.

Test Sample	Number	Test Sample	Number								
Single System		Mixture System	Mass Ratio (%)								
			9:1	8:2	7:3	6:4	5:5	4:6	3:7	2:8	1:9
DFTNAN	A1	DFTNAN/RDX	B1	B2	B3	B4	B5	B6	B7	B8	B9
RDX	A2	DFTNAN/HMX	C1	C2	C3	C4	C5	C6	C7	C8	C9
HMX	A3	DFTNAN/CL-20	D1	D2	D3	D4	D5	D6	D7	D8	D9
CL-20	A4	DFTNAN/TKX-50	E1	E2	E3	E4	E5	E6	E7	E8	E9
TKX-50	A5	/									

**Table 2:** DSC data of DFTNAN at different heating rates.

*β*/°C·min^−1^	*T*^’^_eo_/°C	*T*_p_/°C	*T*_f_/°C
2	246.20	251.64	255.41
5	266.63	272.59	275.76
10	285.45	289.72	295.12
15	291.17	297.99	304.02

**Table 3:** Thermal decomposition kinetic parameters, thermodynamic parameters and thermal safety parameters of DFTNAN.

Kissinger’s Method			Ozawa’s Method		*T*_ASDT_/°C	*T*_b_/°C	Δ*H*^≠^/ kJ·mol^−1^	Δ*S*^≠^/ kJ·mol^−1^·K^−1^	Δ*G*^≠^/ kJ·mol^−1^
*A*_k_/min^−1^	*E*_k_/kJ·mol^−1^	*R* ^2^	*E*_o_/kJ·mol^−1^	*R* ^2^					
4.73 × 10^8^	97.93	0.9977	101.78	0.9980	226.33	249.03	95.28	−92.26	146.13

**Table 4:** Evaluation standards of the compatibility for explosives.

Criteria △*T*_p_ (°C)	Content
<4	-the mixture is considered to be compatible
4–20	-the mixture is considered “moderately” incompatible (it is necessary to apply other methods of determining the compatibility);
>20	-the mixture is considered to be incompatible

**Table 5:** Compatibility results of DFTNAN-based mixture explosives by DSC.

System	Mass Ratios of Mixture	OB/%	*T*_p_/°C	Δ*T*_p_/°C	Compatibility Judgment
DFTNAN	/	−43.01	273	/	/
RDX	/	−21.62	231	/	/
DFTNAN/RDX	90:10	−40.87	240	9	“Moderately” incompatible
	80:20	−38.73	224	7	“Moderately” incompatible
	70:30	−36.59	225	6	“Moderately” incompatible
	60:40	−34.46	227	4	Compatible
	50:50	−32.32	228	3	Compatible
	40:60	−30.18	228	3	Compatible
	30:70	−28.04	227	4	Compatible
	20:80	−25.90	229	2	Compatible
	10:90	−23.76	229	2	Compatible
HMX	/	−21.62	281	/	/
DFTNAN/HMX	90:10	−40.87	245	28	Incompatible
	80:20	−38.73	239	34	Incompatible
	70:30	−36.59	239	34	Incompatible
	60:40	−34.46	240,272	33,9	Incompatible
	50:50	−32.32	239,269	34,12	Incompatible
	40:60	−30.17	277	4	Compatible
	30:70	−28.04	277	3	Compatible
	20:80	−25.90	279	1	Compatible
	10:90	−23.76	279	2	Compatible
CL-20	/	−10.96	237	/	/
DFTNAN/CL-20	90:10	−39.81	210,231	24,42	Incompatible
	80:20	−36.60	207	66	Incompatible
	70:30	−33.40	207	66	Incompatible
	60:40	−30.19	209	63	Incompatible
	50:50	−26.98	209,244	64,7	Incompatible
	40:60	−23.78	208,243	65,6	Incompatible
	30:70	−20.57	239	2	Compatible
	20:80	−17.37	239	2	Compatible
	10:90	−14.16	240	3	Compatible
TKX-50	/	−27.12	240,289	/	/
DFTNAN/TKX-50	90:10	−41.42	179	94	Incompatible
	80:20	−39.83	174	99	Incompatible
	70:30	−38.24	170	103	Incompatible
	60:40	−36.65	169	104	Incompatible
	50:50	−35.06	160	113	Incompatible
	40:60	−33.48	161	112	Incompatible
	30:70	−31.89	160,214,294	113,265	Incompatible
	20:80	−30.30	155,221,294	118,195	Incompatible
	10:90	−28.71	154,225,301	1,191,512	Incompatible

## Data Availability

Data sharing is not applicable to this article.
